# Effects of *Leishmania major* infection on the gut microbiome of resistant and susceptible mice

**DOI:** 10.1007/s00253-024-13002-y

**Published:** 2024-01-19

**Authors:** Jakub Mrázek, Lucie Mrázková, Chahrazed Mekadim, Taťána Jarošíková, Imtissal Krayem, Yahya Sohrabi, Peter Demant, Marie Lipoldová

**Affiliations:** 1 Laboratory of Anaerobic Microbiology, Institute of Animal Physiology and Genetics of the Czech Academy of Sciences, Videnska 1083, 142 20 Prague, Czech Republic; 2 Laboratory of Molecular and Cellular Immunology, Institute of Molecular Genetics of the Czech Academy of Sciences, Videnska 1083, 142 20 Prague, Czech Republic; 3 Faculty of Biomedical Engineering, Czech Technical University in Prague, Namestí Sitna 3105, 272 01 Kladno, Czech Republic; 4 Department of Medical Genetics, 3Rd Faculty of Medicine, Charles University, Ruská 87, 100 00 Prague 10, Czech Republic; 5 Department of Cardiology I-Coronary and Peripheral Vascular Disease, Heart Failure, University Hospital Münster, Westfälische Wilhelms-Universität, Münster, Germany; 6 Department of Molecular and Cellular Biology, Roswell Park Comprehensive Cancer Center, Buffalo, NY 14263 USA

**Keywords:** *Leishmania major* infection, Cutaneous leishmaniasis, Gut microbiota, Microbiome analysis, Mouse models, Host-parasite interaction

## Abstract

**Abstract:**

Cutaneous leishmaniasis, a parasitic disease caused by *Leishmania major*, is a widely frequent form in humans. To explore the importance of the host gut microbiota and to investigate its changes during *L. major* infection, two different groups of mouse models were assessed. The microbiome of two parts of the host gut—ileum and colon—from infected and non-infected mice were characterised by sequencing of 16S rDNA using an Ion Torrent PGM platform. Microbiome analysis was performed to reveal changes related to the susceptibility and the genetics of mice strains in two different gut compartments and to compare the results between infected and non-infected mice. The results showed that *Leishmania* infection affects mainly the ileum microbiota, whereas the colon bacterial community was more stable. Different biomarkers were determined in the gut microbiota of infected resistant mice and infected susceptible mice using LEfSe analysis. Lactobacillaceae was associated with resistance in the colon microbiota of all resistant mice strains infected with *L. major*. Genes related to xenobiotic biodegradation and metabolism and amino acid metabolism were primarily enriched in the small intestine microbiome of resistant strains, while genes associated with carbohydrate metabolism and glycan biosynthesis and metabolism were most abundant in the gut microbiome of the infected susceptible mice. These results should improve our understanding of host-parasite interaction and provide important insights into the effect of leishmaniasis on the gut microbiota. Also, this study highlights the role of host genetic variation in shaping the diversity and composition of the gut microbiome.

**Key points:**

*• Leishmaniasis may affect mainly the ileum microbiota while colon microbiota was more stable.*

*• Biomarkers related with resistance or susceptibility were determined in the gut microbiota of mice.*

*• Several pathways were predicted to be upregulated in the gut microbiota of resistant or susceptible mice.*

**Graphical Abstract:**

**Figure:**
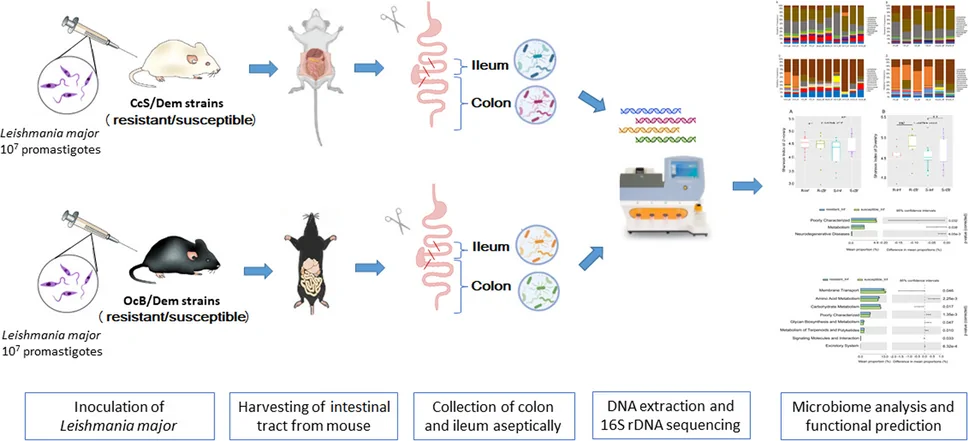

**Supplementary Information:**

The online version contains supplementary material available at 10.1007/s00253-024-13002-y.

## Introduction

The gut harbours a complex and dynamic community of microorganisms called the gut microbiota that plays a crucial role in maintaining individual health and welfare. The healthy intestinal microbiota is a complex ecological community of a huge number of microorganisms comprising, bacteria, viruses, protozoa and fungi. The gastrointestinal tract is a multi-organ system and the colonisation of microbiota differs between intestinal segments providing disparate functions (Martinez-Guryn et al. 2019). Several factors, such as host genetics, age, gender, gastrointestinal infections, antibiotic use, diet and lifestyle, may have an effect on the distribution of the gut microbiota among individuals. Alteration of microbial balance of the gut, or “dysbiosis”, may have a major impact on an individual’s health (Chang and Kao 2019). Many evidences have illustrated that host genetics may contribute in structuring gut microbial communities in human and mice (Bubier et al. 2021; Suzuki et al. 2019; Org and Lusis 2018; Goodrich et al. 2014; Goodrich et al. 2016). However, it is still unclear how host genes regulate the gut microbiota.

The intestinal microbiota has a potential impact on the maturation, development and function of major components of the host’s immune system. In addition, gut microbiota can modulate host immunity, and it may also affect the host immune response against parasites (Partida-Rodríguez et al. 2017). The relationship between parasite and host-microbiota is bi-directional; parasitic infections shape the host-microbial composition directly or indirectly through systemic immune responses. Conversely, host-microbiota predisposes an individual to parasite infection, which could influence disease outcome including leishmaniasis (Bhattacharjee et al. 2017).

Leishmaniasis is a neglected tropical disease. It is caused by *Leishmania*, an obligatory intracellular protozoan parasite, which comprises a group of more than 20 species that are transmitted to humans by the bite of an infected female sand fly vector of the genus *Phlebotomus* in the Old World and the genus *Lutzomyia* in the New World (Alvar et al. 2012; Desjeux 1996). Three main clinical forms of this disease have been described in humans: cutaneous leishmaniasis (CL); mucocutaneous leishmaniasis (MCL) and visceral leishmaniasis (VL) also known as kala-azar (Desjeux 1996; Dedet 2002). Cutaneous leishmaniasis is the most common and less severe form of the disease. According to the WHO, more than 1 million new cases of CL occur annually (Alvar et al. 2012). *Leishmania major* causes cutaneous leishmaniasis in North Africa, the Middle East and Asia (Alemayehu and Alemayehu 2017).

Leishmaniasis manifests differently depending on the infecting species, environmental and social context and the genotype of the mammalian host (Lipoldová and Demant 2006). This disease has several limitations for recovery including suboptimal and inconsistent clinical diagnosis of disease, restricted treatments and development of drug resistance. Therefore, it is essential to apply novel tools and identification methods to understand the pathogenesis better for developing more effective diagnosis and potential treatment. By development of molecular biology and bioinformatics, microbiome analysis revealed the crucial role of host-microbiota and its metabolites in the progression and development of major human diseases as well as in regulating the function of the immune system (Kamada et al. 2013; Allen 2016; Partida-Rodríguez et al. 2017; Wang et al. 2017; Ding et al. 2019).

The role of the cutaneous microbiota during CL has been investigated in several studies, which analysed the association between host cutaneous microbiome and lesion development in human CL (Salgado et al. 2016; Gimblet et al. 2017; Misra and Singh 2019) or by studying the disease manifestation in germ-free mice model and in mice kept in conventional conditions (Vieira et al. 1987, 1998; Oliveira et al. 2005; Borbón et al. 2019). The majority of studies have been focused on the characterisation of the midgut microbiome of different species of vectors of leishmaniasis (Naik et al. 2012; Dostálová and Volf 2012; Monteiro et al. 2016, 2017; Karakuş et al. 2017; Fraihi et al. 2017; Kelly et al. 2017; Karimian et al. 2019; Vivero et al. 2019; Gunathilaka et al. 2020; Campolina et al. 2020). A limited number of studies have been conducted to examine the correlation between *Leishmania* and the host’s gut microbiota. Few studies have reported the effect of the indigenous microbiota on the regulation of immune response during *Leishmania* infection highlighting how the interaction between the host immune system and the microbiota can determine the outcome of leishmaniasis in the murine model (Oliveira et al. 1999, 2005; Julia et al. 2000; Lamour et al. 2015; Lopes et al. 2016; Campolina et al. 2020). In *L. major* infection models, results have demonstrated that Swiss/NIH germ-free infected mice were unable to heal lesions and had high numbers of parasitised macrophages in comparison to their conventional counterparts (Oliveira et al. 1999). Despite this, cells from these mice produced high levels of IFNγ and generated a strong Th1 immune response (Oliveira et al. 2005). These observations suggest that the microbiota act as an adjuvant for macrophages to efficiently respond to this intracellular parasite infection, which strongly elucidates that the gut microbiota is implicated in establishing a strong host immune response to the parasite (Oliveira et al. 1999, 2005). Furthermore, it was reported that iNOS + macrophage numbers were reduced in germ-free mice infected by *L. major*. The ability of germ-free mice to control the infection was established when they were colonised by microbiota from conventional mice. This illustrates the critical role of host-microbiota in the innate immune response to *L. major* infection by driving host macrophages to develop a resistance phenotype (Lopes et al. 2021). Moreover, Lamour et al. (2015) revealed that the differences in leishmaniasis outcome between the two different mouse strains, C57BL/6 self-healing and BALB/c non-healing mice, are associated with host faecal microbiome and host metabolism profiles. They concluded that a successful defence against *Leishmania* infection may be due to an efficient connection between immunological, metabolic and gut microbial systems (Lamour et al. 2015). Another study has reported that LACK-specific T cells in the infection of BALB/c mice with *L. major* acquired the ability to rapidly secrete interleukin 4 (IL-4) after they were primed in gut-associated lymphoid tissues by cross-reactive microbial Ags (antigens) derived from the indigenous intestinal microbiota (Julia et al. 2000). Recently, the only study, which analyses the correlation between leishmaniasis and human microbiota, has been performed by Lappan and colleagues (Lappan et al. 2019). They used a meta-taxonomic approach to determine the prokaryotic and eukaryotic composition of faecal samples of individuals from an endemic region for VL in India caused by *Leishmania donovani* and compared the differences in microbial composition between VL cases and non-VL endemic controls (EC) (Lappan et al. 2019). No significant difference in the diversity of prokaryotic or eukaryotic microflora was observed between VL cases and EC. However, some differences were reported at the individual bacterial level or protozoan taxa. The level of *Ruminococcaceae* UCG- 014 and *Gastranaerophilales_*uncultured bacterium was higher in EC compared to VL cases. In addition, *Pentatrichomonas hominis* was more abundant in VL cases than in EC, whereas it was the reverse for *Entamoeba* (Lappan et al. 2019). According to our knowledge, no studies on the role of different compartments of host gut microbiota on leishmaniasis progress has been performed, nor the influence of *L. major* infection on the gut microbiota composition in its different compartments.

In the present study, microbiome analysis of two parts of the digestive tract—ileum and colon—of *L. major*-infected/non-infected mice were assessed in order to (i) characterise the microbiota composition of two compartments of murine models, (ii) to investigate whether the microbiota composition of two compartments differs between *L. major*-infected and non-infected mice, and/or between resistant and susceptible strains, (iii) to determine the effects of *L. major* on the microbial diversity on host gastrointestinal tract and (iv) to reveal the potential influence of host genetic on gut microbiota after *L. major* infection.

To achieve this, we analysed non-infected and *L. major*-infected eight mouse strains that were derived from two genetically distant, but internally related groups: CcS/Dem (BALB/c, STS, CcS-5, CcS-12, CcS-20) and OcB/Dem (O20, C57BL/10 [B10], C57BL/10-*H2*^*pz*^ [B10.O20]). Each CcS/Dem strain contains a different, random set of approximately 12.5% genes of the donor strain STS and approximately 87.5% genes of the background strain BALB/c (Demant and Hart 1986; Stassen et al. 1996). B10.O20 carries 3.6% of genes of the strain O20 on the B10 genetic background (Krayem et al. 2022). Strains were classified as resistant or susceptible according to their organ pathology and parasite load in organs (Lipoldová et al. 2002; Sohrabi et al. 2018). Highly susceptible strains BALB/c and CcS-12 exhibit massive parasite infiltration into organs and develop large skin lesions, splenomegaly and hepatomegaly. CcS-12 contains even higher parasite numbers in lymph nodes than BALB/c (Lipoldová et al. 2002). Susceptible strain B10.O20 has rather a high parasite load in the skin and develops skin lesions (Sohrabi et al. 2018). Resistant strains STS, O20, B10 and CcS-5 contain low numbers of parasites in their organs but do not develop skin lesions, splenomegaly or hepatomegaly (Lipoldová et al. 2002; Sohrabi et al. 2018). Intermediate strain CcS-20 has intermediate numbers of parasites in skin, develops none or small lesions and does not exhibit splenomegaly or hepatomegaly (Lipoldová et al. 2002; Sohrabi et al. 2018).

## Material and methods

### Mice

Females of mice strains BALB/c (9 infected; 9 non-infected), STS (9 infected; 9 non-infected), CcS-5 (8 infected, 8 non-infected), CcS-12 (4 infected, 6 non-infected), CcS-20 (9 infected, 9 non-infected), O20 (8 infected, 10 non-infected), C57BL/10 [B10] (3 infected, 3 non-infected) and C57BL/10-*H2*^*pz*^ [B10.O20] (13 infected, 14 non-infected) were used in this experiments. Their age was between 8 and 16 weeks at the time of infection. The strains were classified previously according to organ pathology, systemic disease and parasite load in organs (Sohrabi et al. 2018; Lipoldová et al. 2002). Mice were maintained on a 12-h light/dark cycle in specific-pathogen-free (SPF) conditions.

The animals were monitored for skin lesions and body weight development for another 8 weeks and then euthanised. The size of the skin lesions was measured weekly using the Profi LCD Electronic Digital Caliper Messschieber Schieblehre Messer (Shenzhen Xtension^2^ Technology Co., Ltd. Guangdong, China), which has an accuracy of 0.02 mm. The digestive tract was removed, and ileum and colon parts were separated under sterile conditions into a sterile microcentrifuge tube and stored immediately at − 80 °C until DNA extraction.

### Parasite

Sub strain of *L. major* LV 561 (MHOM/IL/67/LRCL 137 JERICHO II) used in the current experiments is kept in liquid nitrogen in the Institute of Molecular Genetics. It was maintained in rump lesions of BALB/c females. Flagella-free amastigotes were isolated from BALB/C lesions and transformed to promastigotes using blood agar SNB-9 (Grekov et al. 2011). 10^7^ flagellate promastigotes from the second passage cultivated for 6 days were inoculated in 50 µl sterile saline then injected subcutaneously into a mice rump (Lipoldová et al. 2000). Control non-infected mice were injected with 50 µl sterile saline.

### DNA extraction

The DNA was extracted from the samples (ileum and colon) using QIAamp PowerFecal DNA Kit (Qiagen, Hilden, Germany) as per the manufacturer’s instructions. The disintegration step was performed with a FastPrep-24 homogenisation system (MP Biomedicals, Santa Ana, CA, USA) for 1 mn at a maximum speed of 6.5 m/s. The elution was done with 60 μL of elution buffer. The eluted DNA was stored at − 20 °C until further use.

### Preparation of 16S rDNA and sequencing

The hypervariable V4-V5 region of the 16S rRNA gene was amplified from the total bacterial extracted DNA. Then, the purified amplicons (300 bp) were processed as previously described (Mekadim et al. 2022). Briefly, libraries were prepared using NEBNext Fast DNA Library Prep Set kit (New England Biolabs, Ipswich, MA, USA) according to Milani et al. (2013). The sequencing was then performed on an Ion Torrent platform (Thermo Fisher Scientific, Waltham, MA, USA).

### Microbiome and statistical analysis

Bacterial 16S rDNA sequences were obtained in FASTQ format, and they were analysed by the QIIME 2 2020.2 pipeline (Bolyen et al. 2019). The quality control, filtration and trimming were performed on the demultiplexed sequences using DADA2 (Callahan et al. 2016). Clustering and taxonomy classification was assigned to the resulting amplicon sequence variants (ASVs) with VSEARCH using SILVA database release132 with 99% OTU (operational taxonomic unit) reference sequences (Rognes et al. 2016).

Shannon index of diversity (alpha diversity) was determined based on the Kruskal–Wallis test, and principal coordinate analysis (PCoA) based on Bray–Curtis distance (beta diversity) was estimated after samples were rarefied. Alpha diversity box plots for Shannon index of diversity and the two-dimensional PCoA plots were generated by qiime2R (Bisanz 2018) (https://github.com/jbisanz/qiime2R) and ggplot2 (Wickham 2016) (https://ggplot2.tidyverse.org) packages in R-Studio (version 3.6.3) (RStudio Team 2020), (http://www.rstudio.com/). Ellipses mark 95% of confidence around each group, and a *p*-value ≤ 0.05 was considered statistically significant. The impact of certain factors (susceptibility-infection, host genetics) on the relative composition of microbiomes was evaluated using the *adonis* plugin in QIIME2, utilising Adonis permutational multivariate analysis (Adonis/PERMANOVA) and Bray–Curtis distance matrix to evaluate the dissimilarity among samples with permutation set at 999 (Anderson 2017).

The linear discriminant analysis (LDA) with effect size (LefSe) algorithm (Segata et al. 2011) in Galaxy module http://huttenhower.sph.harvard.edu/galaxy was used for biomarker detection based on the factorial Kruskal–Wallis (KW) test and the pairwise Wilcoxon test to detect taxa with significant differential relative abundances of bacterial families in the susceptible and infected resistant group in colon and ileum with alpha values of 0.05 and a threshold value of 2.0 on the logarithmic linear discriminant analysis (LDA) scores for discriminative features.

PICRUSt 2 was applied for metabolic functional prediction (Langille et al. 2013). The predictions were collapsed into KEGG pathways at levels 2 and 3, and the resulting abundance table was imported in the STAMP v2.1.3 program for statistical analysis (Parks et al. 2014) by using non-corrected Welch’s *t*-test type two-sided, with the confidence interval (CI) method of Welch’s inverted adjustment of 0.95 between resistant and susceptible strains (*p* < 0.05) considered to be of statistical significance. The relationships among functional capacities were analysed by PCA (principal component analysis).

## Results

### Impact of *Leishmania* infection on gut microbiome diversity 

A total of 13,671,855 sequences were obtained from samples of different mice. The majority of these were from the colon part (9,762,045 sequences). The mean sequence length was 260 bp. The analysis of two mice strain groups CcS/Dem and OcB/Dem was done independently in which the ileum and colon samples were treated separately.

**Fig. 1:**
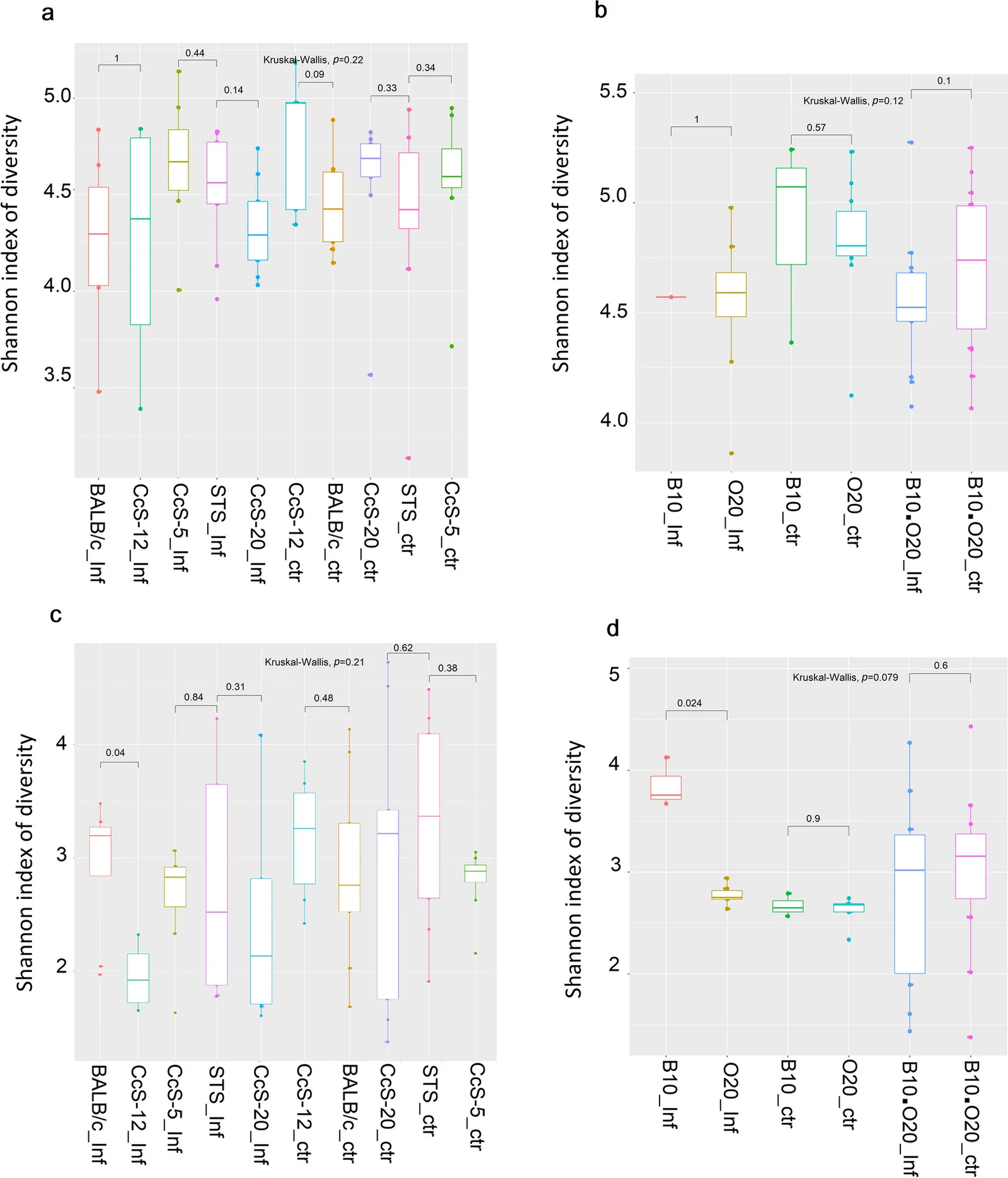
Microbiome alpha diversity (Shannon index) among different mouse strains (susceptibles/ resistants) of infected or non-infected mice in **a** colon of CcS/Dem, **b** colon of OcB/Dem, **c** ileum of CcS/Dem and **d** ileum of OcB/Dem. *p*-value ≤ 0.05 was considered statistically significant. [Inf infected, ctr control (non-infected)]

Alpha diversity of samples from different gastrointestinal segments (ileum and colon) of different mouse strains was evaluated to determine the bacterial diversity of each animal group (susceptible/resistant and infected/ non-infected). The Shannon index was commonly used to determine the diversity and abundance of species in a community. The results of the Shannon index were represented in the boxplot graph (Fig. 1, Supplemental Fig. S1). The different values were reported in Supplemental Table S1. The bacterial community was the largest and most diverse in the colon and least diverse in the ileum (Fig. 1). The diversity of the bacterial community was more stable in the CcS/Dem group than in the OcB/Dem group. For CcS/Dem strains, the results showed non-significant differences (*p* > 0.05) between infected and non-infected animals or between resistant and susceptible mice in both ileum and colon microbiome (Fig. 1a, c) (Supplemental Table S1). For OcB/Dem strains, a significant difference was noticed between infected and non-infected resistant mice in the colon (*p* = 0.02) and the ileum (*p* = 0.01) microbiome (Fig. 1b, d) (Supplemental Table S1). The diversity of infected mice was lower than that of non-infected mice in the colon of the OcB/Dem group (Fig. 1b). Non-significant statistical differences were observed between different mice strains in the colon microbiome of all animals (Supplemental Figs. S1a, b, Supplemental Table S1). The diversity in the ileum microbiome of the susceptible BALB/c strain was significantly higher (*p* = 0.04) than in the infected susceptible CcS-12 strain (Supplemental Fig. S1c). The diversity in the ileum microbiome was significantly higher (*p* = 0.02) in the infected resistant B10 strain than in the infected resistant O20 strain (Supplemental Fig. S1d).

**Fig. 2:**
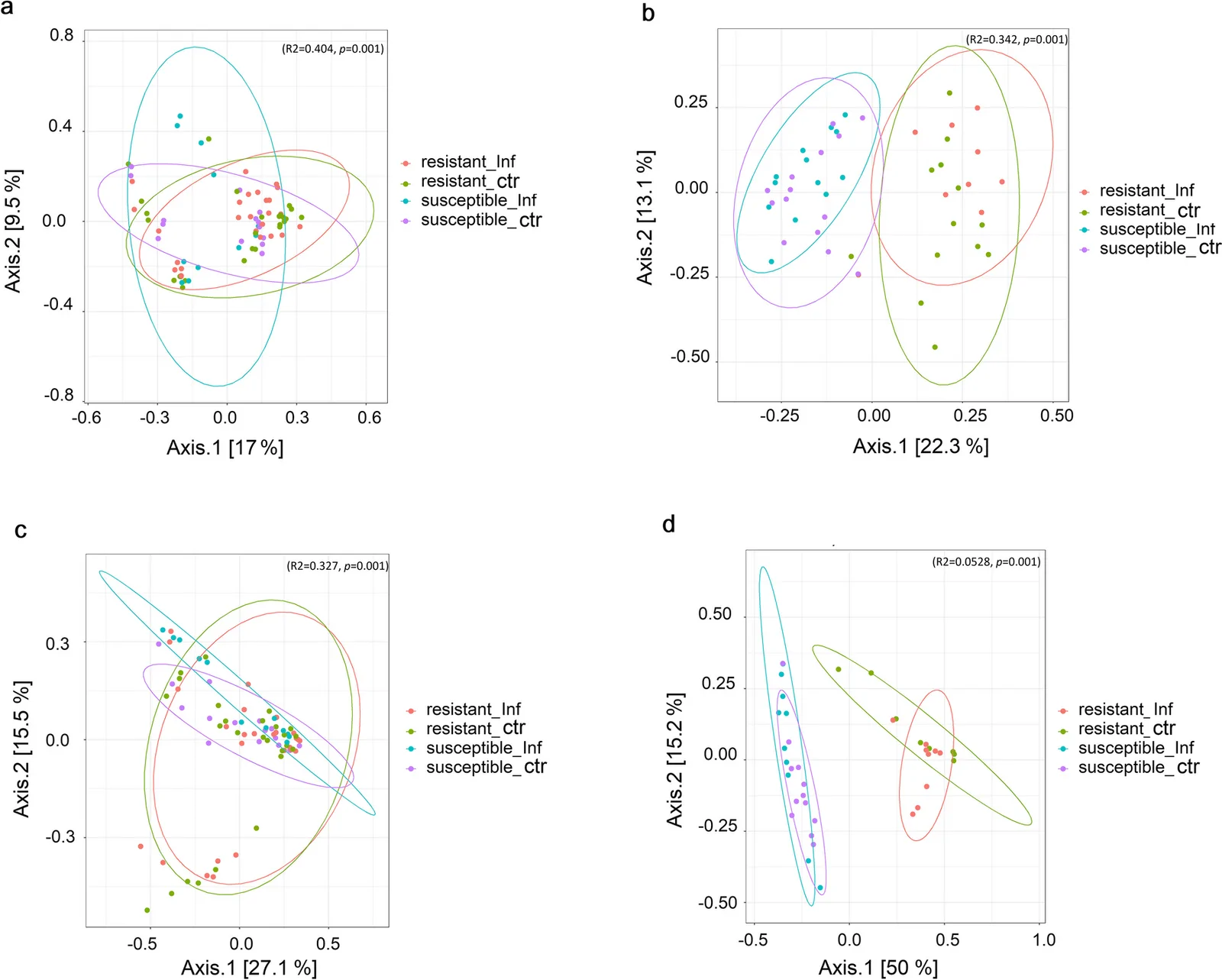
Principal coordinate analysis (PCoA) plots based on the Bray–Curtis distance showed distinct clusters among different groups (susceptibles/ resistants) of infected or non-infected mice in: a) colon of CcS/Dem, b) colon of OcB/Dem, c) ileum of CcS/Dem and d) ileum of OcB/Dem. Ellipses mark 95% confidence ellipses around each group and (*p*-value ≤ 0.05) was considered statistically significant. [Resistant_Inf: infected resistant, Resistant_ctr: resistant control (non-infected), Susceptible_Inf: infected susceptible, Susceptible_ctr: susceptible control (non-infected)]

In order to study the similarity between different samples, beta diversity was used to analyse the composition between bacterial communities in the colon and ileum separately. Principal coordinate analysis (PCoA) based on Bray–Curtis distance was performed to compare the microbiome diversity between resistant vs susceptible and infected vs non-infected mice from different strains (Fig. 2). The diversity of colon microbiome was largely dispersed in samples from CcS/Dem strains (*R*^2^ = 0.404, *p* = 0.001) (Fig. 2a). In the ileum microbiome of samples from CcS/Dem strains, beta diversity was more centred in susceptible mouse strains and dispersed in resistant mouse strains (*R*^2^ = 0.327, *p* = 0.001) (Fig. 2c).

**Fig. 3:**
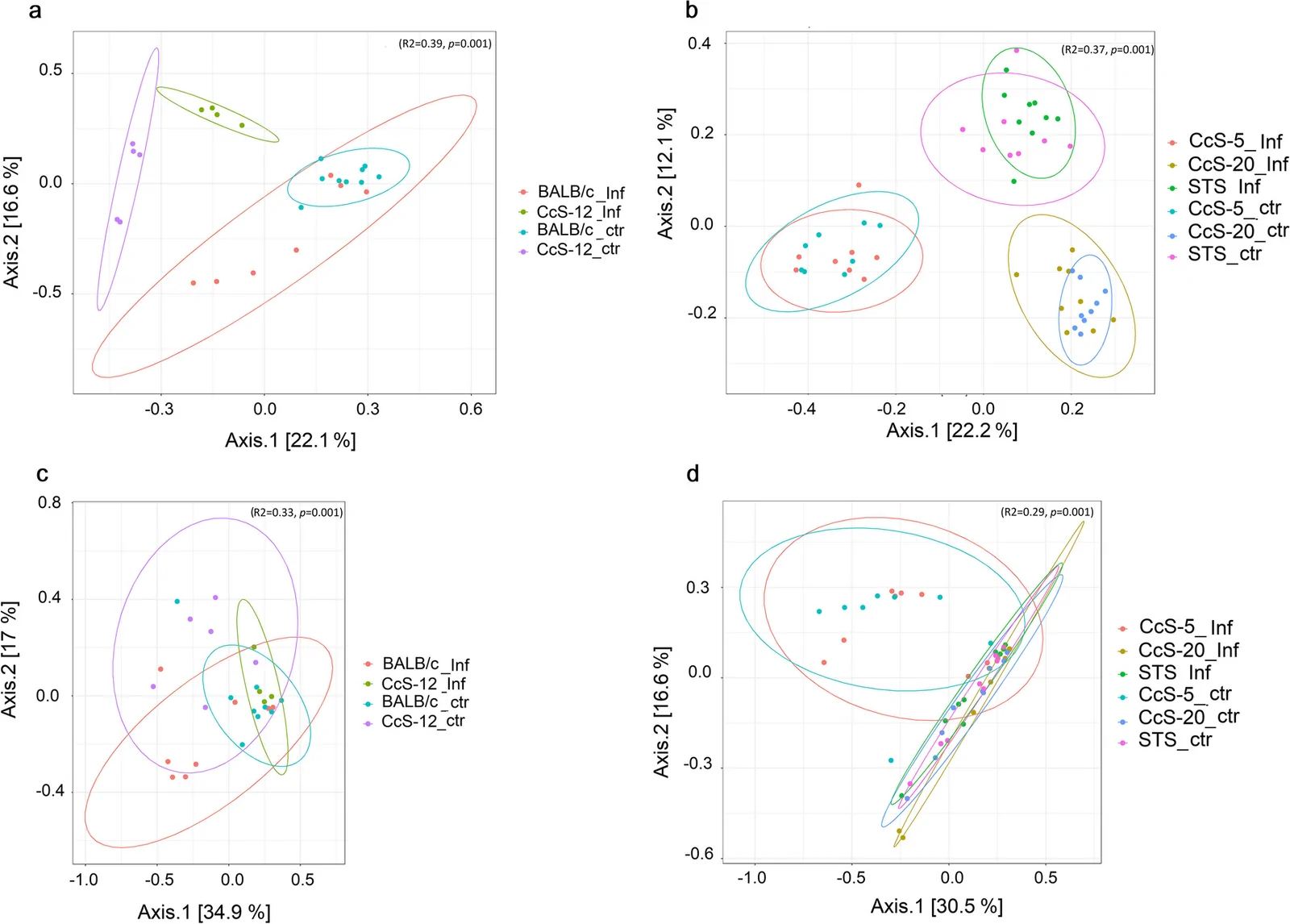
Principal coordinate analysis (PCoA) plots based on the Bray–Curtis distance showed distinct clusters among infected or non-infected mouse strains of CcS/Dem group in **a** colon of susceptible CcS/Dem, **b** colon of resistant CcS/Dem, **c** ileum of susceptible CcS/Dem and **d** ileum of resistant CcS/Dem. Ellipses mark 95% confidence ellipses around each group, and *p*-value ≤ 0.05 was considered statistically significant. [Inf infected, ctr control (non-infected)]

Beta diversity of different strains from CcS/Dem mouse series was analysed separately (Fig. 3).

In the colon microbiome, a higher significant difference was observed between susceptible mouse strains (*R*^2^ = 0.39, *p* = 0.001) and resistant mouse strains (*R*^2^ = 0.37, *p* = 0.001), and clusters of different strains were distinguished and separated to each other (Fig. 3a, b). However, in the ileum microbiome, all resistant samples were clustered together separated to CcS-20 strain (*R*^2^ = 0.29, *p* = 0.001) (Fig. 3d). The results showed that beta diversity was similar in the infected and non-infected mice except samples from the CcS-12 susceptible strain where they were separated into an infected group and a non-infected group in both colon and ileum microbiome (*R*^2^ = 0.33, *p* = 0.001) (Fig. 3a, c).

Moreover, for OcB/Dem mice, in both colon and ileum microbiomes, two major clusters were distinguished: a cluster of resistant mice and the other cluster for susceptible ones (*R*^2^ = 0.342, *p* = 0.001) (Fig. 2b, d). However, there were no significant differences between infected and non-infected mice, except in the ileum microbiome of resistant lines, the clusters of infected and non-infected mice were separated (*R*^2^ = 0.528, *p* = 0.001) (Fig. 2d).

### Impact of *Leishmania* infection on gut microbiota composition

#### Colon microbiota

**Fig. 4:**
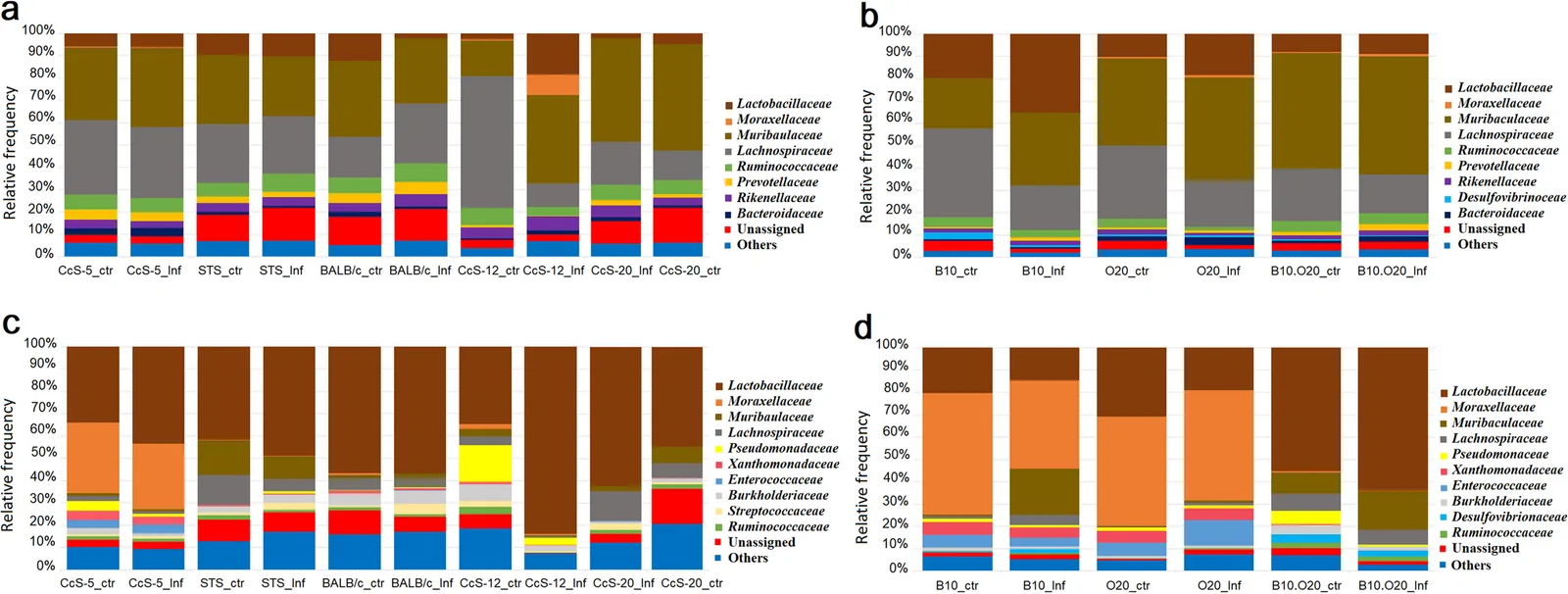
Relative abundance of the microbial population at the family level among different infected or non-infected mouse strains in **a** colon of CcS/Dem, **b** colon of OcB/Dem, **c** ileum of CcS/Dem and **d** ileum of OcB/Dem. [Inf infected, ctr control (non-infected)]

In general, gut bacterial abundancy was variable and dissimilar in different mouse strains. For the CcS/Dem mice group (Supplemental Table S2, Fig. 4a, Supplemental Fig. S2a)**,** the microbiota of colon samples from all animals was dominated by Bacteroidetes and Firmicutes (Supplemental Fig. S2a). *Bacteroidetes* was slightly higher in the infected susceptible CcS-12 strain (49.2%) and Muribaculaceae was the most abundant family (39.5%). Muribaculaceae was the dominant family in infected intermediate strains CcS-12 (46.39%). Lachnospiraceae was more abundant in the infected resistant CcS-5 strain (31.9%) than the infected susceptible CcS-12 strain (10.3%). However, Lachnospiraceae was the dominant bacterial family in the colon microbiome of the non-infected CcS-12 strain (59.1%) (Fig. 4a).

**Fig. 5:**
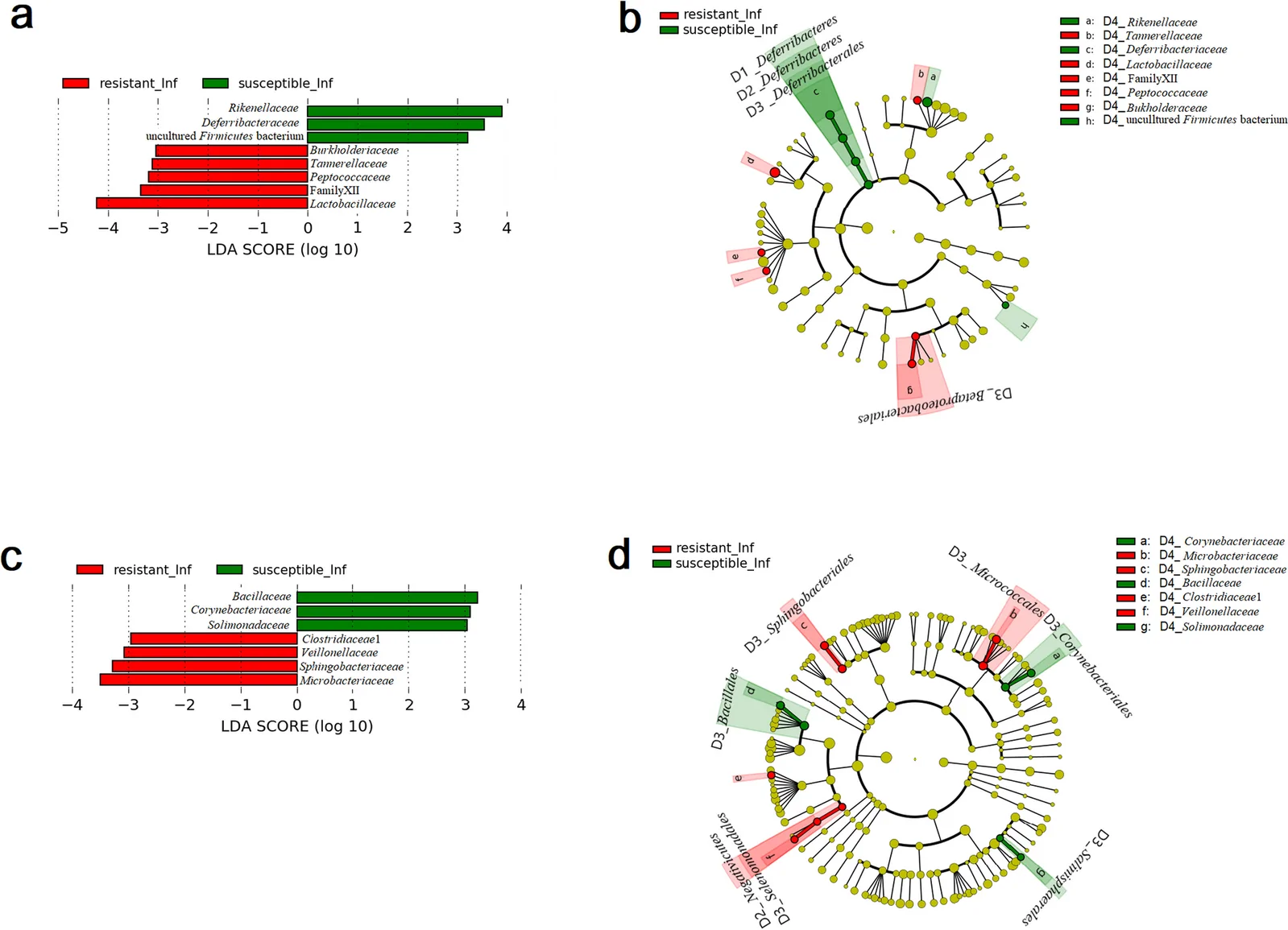
Linear discriminant analysis (LDA) effect size (LEfSe) of taxa at family level in infected susceptible CcS/Dem mice (in green) and infected resistant CcS/Dem mice (in red) in **a**, **b** colon and **c**, **d** ileum with alpha values of 0.05 and a threshold value of 2.0 (**b** and **d** in cladogram present a phylogenetic plot of LEfSe and taxa: D1 for Phylum level, D2 for Class level, D3 for Order level and D4 for Family level). [Resistant_Inf infected resistant, Susceptible_Inf infected susceptible]

LEfSe analysis identified Lactobacillaceae as the highest biomarker correlated with the colon microbiota of infected resistant CcS/Dem mice group in addition to Family XIII (Clostridiales), Peptococcaceae, Tannerellaceae and Burkholderiaceae. Rikenellaceae, Deferribacteriaceae and a family of uncultured Firmicutes bacterium were identified as biomarkers associated with the infected susceptible CcS/Dem mice group in their colon (Fig. 5a, b).

For OcB/Dem mouse strains (Supplemental Table S3, Fig. 4b, Supplemental Fig. S2b)**,** the colon microbiota of all animals was dominated by Bacteroidetes and Firmicutes phyla (Supplemental Fig. S2b). Bcteroidales was the dominant order in all animals, but it was slightly higher (61.8%) in the infected susceptible group (B10.O20) where Muribaculaceae was the most abundant family (52.9%). The relative abundance of Lactobacillaceae and *Lactobacillus* was higher (35.2%) in the infected resistant B10 mice strain than in the infected susceptible group (B10.O20) (8.9%). Lachnospiraceae was found in all animals, but it was estimated slightly higher in the non-infected resistant B10 mice strain (39.8%) (Fig. 4b).

LEfSe results (Fig. 5a, b) identified two bacterial families (Lactobacillaceae and Clostridiaceae1) that were related with the infected resistant OcB/Dem mice group, while Ruminococcaceae, Rikenellaceae, Moraxellaceae, Tennerellaceae and Christensenellaceae were associated with the infected susceptible OcB/Dem mice group.

#### Ileum microbiota

Globally, Firmicutes, Proteobacteria and Bacteroidetes were the main phyla in the ileum microbiota of all mice. For the CcS/Dem mice group (Supplemental Table S4, Fig. 4c, Supplemental Fig. S2c), Firmicutes was the abundant phylum in the ileum microbiota of infected resistant animals where the frequency was 52.3% in the strain CcS-5 and 61.4% in the strain STS. Firmicutes was higher and the dominant phylum in the ileum microbiota of infected susceptible mice with percentages of 86.3% in the CcS-12 strain, 69.7% in the BALB/c strain and in the infected intermediate strain CcS-20 81.2%. Proteobacteria was higher (38.2%) in the ileum microbiota of the infected resistant strain CcS-5. Bacteroidetes was higher (12.9%) in the ileum microbiota of the infected resistant strain STS (Supplemental Fig. S2c). Gammaproteobacteria was higher (36.1%) in the ileum microbiota of the infected resistant strain CcS-5, in which the family of Moraxellaceae and the genus of *Acinetobacter* (29.5%) were higher in this bacterial class. However, the family of Moraxellaceae and the genus of *Acinetobacter* were infrequent in ileum microbiome of the resistant strain STS. Lactobacillaceae and *Lactobacillus* were the most abundant family and genus, respectively, in the infected resistant mice with percentages of 43.4% for the strain CcS-5 and 49.0% for the strain STS. Lactobacillaceae and *Lactobacillus* were higher and the dominant family and genus, respectively, in the susceptible and infected intermediate animals with percentages of 83.8% in the strain CcS-12, 56.9% in the strain BALB/c and 62.36% in the strain CcS-20. Muribaculaceae was higher in resistant STS mice with a percentage of 10.1% in ileum microbiota of infected STS mice and 15.7% in ileum microbiota of non-infected ones (Fig. 4a).

LEfSe results (Fig. 5c, d) indicated four bacterial families were related with resistance in ileum microbiota of infected CcS/Dem mice (Microbacteriaceae, Sphingobacteriaceae, Veillonellaceae and Clostridiaceae1) and three bacterial families (Bacillaceae, Corynebacteriaceae and Solimonadaceae) in ileum microbiota of infected susceptible CcS/Dem series.

For the OcB/Dem mouse group (Supplemental Table S5, Fig. 4d, Supplemental Fig. S2d)**,** the ileum microbiota of infected resistant animals was dominated by Proteobacteria with relative percentages of 48.3% and 57.8% for the B10 strain and the O20 strain, respectively (Supplemental Fig. S2d). Gammaproteobacteria was the most abundant class in the ileum microbiota of resistant strains represented by 39.5% of Moraxellaceae and *Acinetobacter* in B10-infected mice and 49.4% in O20-infected mice (Fig. 4d). Bacteroidetes was higher in the infected resistance B10 mice (22.1%) than in the non-infected B10 strain (2.6%) and the infected resistance O20 strain (3.2%). The ileum microbiota of infected susceptible B10.O20 mice was mostly dominated by Firmicutes with relative frequencies of 66.4% for non-infected mice and 73.1% for infected mice (Supplemental Fig. S2d). Lactobacillaceae was the most abundant family (63.9%) in the ileum microbiota of infected susceptible B10.O20 mice represented by the genus *Lactobacillus*. Bacteroidetes was higher (17.8%) and Proteobacteria was lower (6.1%) in the ileum microbiota of infected susceptible B10.O20 mice. Muribaculaceae was higher in ileum microbiota of infected susceptible mice (17.4%) and of infected resistant B10 (20.52%) than in the infected resistant O20 strains (0.7%) (Fig. 4d).

**Fig. 6:**
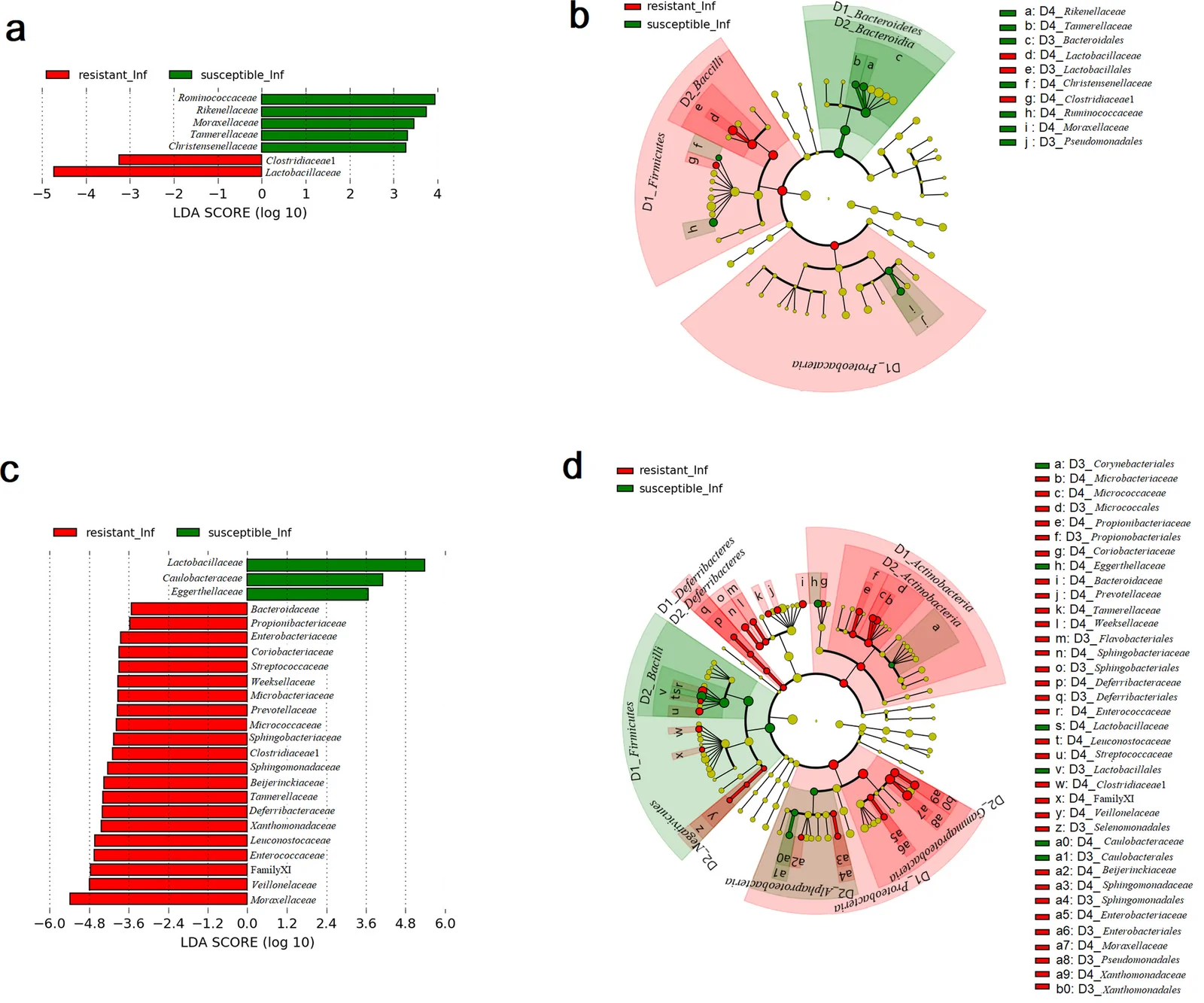
Linear discriminant analysis (LDA) effect size (LEfSe) of taxa at family level in infected susceptible OcB/Dem mice (in green) and infected resistant OcB/Dem mice (in red) in **a**, **b** colon and **c**, **d** ileum with alpha values of 0.05 and a threshold value of 2.0 (**b** and **d** in cladogram present a phylogenetic plot of LEfSe and taxa: D1 for Phylum level, D2 for Class level, D3 for Order level and D4 for Family level). [Resistant_inf infected resistant, Susceptible_inf infected susceptible]

LEfSe results (Fig. 6c, d) from ileum samples showed that various bacterial families were associated with the infected resistant OcB/Dem mice group: Moraxellaceae, Veillonellaceae, Family XI (Clostridiales), Enterococcaceae, Leuconostocaceae, Xanthomonadaceae, Deferribacteriaceae, Tannerellaceae, Beijerinckiaceae, Sphingomonadaceae, Clostridiaceae1, Sphingobacteriaceae, Micrococcaceae, Prevotellaceae, Microbacteriaceae, Weeksellaceae, Streptococcaceae, Coriobacteriaceae, Enterobacteriaceae, Propionibacteriaceae and Bacteroidaceae. Lctobacillaceae, Caulobacteraceae and Eggerthellaceae were associated with the ileum microbiota of the infected susceptible OcB/Dem mice group.

### Impact of host genetics on gut microbiome of non-infected mice

There was no significant difference in alpha diversity between different non-infected mice models used in this study (Supplemental Tables S1 and S2). However, Bray–Curtis dissimilarity was significantly correlated with the genetic make-up of the host (*p* = 0.001) (Fig. 2). Relative abundancy of taxa at different bacterial taxonomical levels was wide-ranging within different mouse strains (Fig. 3, Supplemental Fig. S2, Supplemental Tables S3 and S4).

### Metagenomic functional prediction

Functional inference analyses based on PICRUSt were performed on the microbiota of colon and ileum separately to determine whether there were significant differences in the abundance of potential functions between the two groups of infected mice: resistant and susceptible. Histograms of the relative abundance of functions were generated (Supplemental Fig. S3). The results revealed that the functional genes of the two groups were mainly involved in “cellular processes”, “environmental information processing”, “genetic information processing”, “human diseases”, “metabolism” and “organismal systems” in KEGG level 1 (Supplemental Tables S6-S8).

By collapsing the data at KEGG level 2, the largest difference between the two groups was found in ileum samples of OcB/Dem strains where 31 differently abundant pathways indicated a significant difference in the functions of the infected resistant strains compared with the infected susceptible strains (Supplemental Fig. S2a). The functional prediction showed that the ileum microbiota of infected resistant OcB/Dem strains was principally related to “xenobiotics biodegradation and metabolism”, “amino acid metabolism”, “lipid metabolism” and “cellular processes and signalling”. “replication and repair”, “translation”, “nucleotide metabolism”, “carbohydrate metabolism”, “transcription”, “genetic information processing energy metabolism”, “metabolism of cofactors and vitamins” and “enzyme families” were the most significant pathways associated with the ileum microbiome of infected susceptible OcB/Dem strains (Supplemental Fig. S2a). However, only nine KEGG pathways were significantly different between the two groups in their colon microbiome of OcB/Dem strains (Supplemental Fig. S2b). The majority of predicted functions were significantly abundant in the infected susceptible B10O20 strains: “glycan biosynthesis and metabolism”, “transport and catabolism”, “biosynthesis of other secondary metabolites”, “metabolism”, “enzyme families”, “endocrine system”, “neurodegenerative diseases” and “digestive system”. “Environmental adaptation” was the only significantly abundant function associated with the colon microbiome of resistant OcB/Dem strains (Supplemental Fig. S2b).

The functional prediction in the ileum microbiome of CcS/Dem strains revealed 8 KEGG pathways that were significantly abundant (Supplemental Fig. S2c). The abundance of “membrane transport” and “carbohydrate metabolism” were significantly higher in the ileum microbiome of infected susceptible CcS/Dem mice. Though, the significant abundance of “amino acid metabolism”, “poorly characterised” functions, “glycan biosynthesis and metabolism” and “metabolism of terpenoids and polyketides” was observed in the ileum microbiome of infected resistant CcS/Dem mice (Supplemental Fig. S2c). Only three KEGG pathways were predicted that were significantly different in the colon microbiome of the CcS/Dem strains. All three functions “poorly characterised” functions, “metabolism” and “neurodegenerative diseases” were significantly higher in the colon microbiome of infected susceptible CcS/Dem strains (Supplemental Fig. S2d). Details can be found in the Supplementary Materials (Supplemental Tables S6–S8).

PCA revealed that the functions of ileum microbiota from the same mouse group (infected resistant or infected susceptible) were clustered together (Supplemental Fig. S4). Two clusters were more separated in the ileum of OcB/Dem mice strains with the first two components explaining a total of 92.7% of the variation (Supplemental Fig. S4d). The results suggested that the functional KOs (KEGG orthology) varied mainly in the ileum microbiota (Supplemental Fig. S3c, d). There was no significant difference between the two mice groups (infected resistant and infected susceptible) in the predicted functions of colon microbiota for both mice strains (Supplemental Fig. S3a, b).

## Discussion

Different bacterial communities are harboured along the mice gastrointestinal tract, including stomach, duodenum, jejunum, ileum, cecum, colon and faeces (Gu et al. 2013; Wang et al. 2019; Han et al. 2020). The results from Oliveira et al. (1999) show the importance of the intestinal microbiota for effective resistance to infection with *L. major*. The authors found that germ-free mice infected with *L. major* had significantly larger lesions than the conventional controls. Therefore, the present study was carried out to investigate the microbiome and bacterial diversity in the ileum and colon of two groups of mouse models of leishmaniasis using a next-generation sequencing approach.

The data showed that the intestinal microbiota of OcB/Dem mice was more variable after infection in comparison to the intestinal microbiota of CcS/Dem strains, which was more stable after infection (Figs. 1, 2, and 3, Supplemental Fig. S1, Supplemental Tables S1 and S4). The bacterial composition of the gastrointestinal tract of the two mouse groups was diverse and distinct in different taxonomical levels. Three main phyla were abundant in the gut of all mouse strains: Firmicutes, Bacteroidetes and Proteobacteria. Similar findings were reported in previous studies (Gu et al. 2013; Han et al. 2020). Proteobacteria was the dominant phylum in the ileum microbiota of resistant mice OcB/Dem strains. Firmicutes was the most abundant phylum in the ileum of susceptible mice in OcB/Dem strains and colon of resistant OcB/Dem strains and the ileum and colon of all CcS/Dem strains. Bacteroidetes was higher in the colon of infected susceptible CcS/Dem strains and was the dominant phylum in the colon of susceptible OcB/Dem mice and colon of infected resistant OcB/Dem mice. The ileum was the most affected gastrointestinal part (Figs. 2 and 3), and colon microbiota was more stable. Many findings have indicated more stable microbial communities in the large intestine compared with the small intestine (Gu et al. 2013; Wang et al. 2019; Han et al. 2020). Also, no significant difference was observed in human stool microbiome diversity of visceral leishmaniasis (VL) cases compared to endemic controls (EC) (Lappan et al. 2019).

Using LEfSe analysis, some biomarkers were determined in the gut microbiota of infected resistant mice and infected susceptible mice (Figs. 4 and 5). Clostridiaceae1, Sphingobacteriaceae, Veillonellaceae and Microbacteriaceae were common biomarkers correlated with infected resistant animals in the ileum microbiota. Moraxellaceae, a bacterial family, which belong to the Gammaproteobacteria class, was the highest biomarker in the ileum microbiota of infected resistant OcB/Dem strains, but it was not identified as a biomarker for infected resistant CcS/Dem strains. Accordingly, the Gammaproteobacteria class was correlated only with resistance to *L. major* infection in C57BL/6 (Lamour et al. 2015) and the O20 strain (this paper). Bacilli were associated with the susceptibility in the ileum microbiota of infected OcB/Dem mouse strains. Similar to our finding, a study of the faecal microbiota of the two experimental models of *L. major* infection BALB/c and C57BL/6 found that Gammaproteobacteria was strongly related with the self-healing strain C57BL/6, and the Bacilli class was associated with the non-healing phenotype BALB/c (Lamour et al. 2015). Rikenellaceae was the common biomarker in the colon microbiota of all infected susceptible mouse strains. Tannerellaceae was associated with resistance to leishmaniasis in the colon microbiota of infected resistant CcS/Dem mice and the ileum microbiota of infected OcB/Dem strains. However, it was significantly abundant in the colon microbiota of infected susceptible OcB/Dem strains. Also, Clostridiaceae was significantly abundant in the colon microbiota of infected resistant OcB/Dem strains and the ileum microbiota of all infected resistant strains. The Clostridia class was associated with the non-healing phenotype (Lamour et al. 2015). Lactobacilaceae was correlated with the resistance in the colon microbiota of all infected resistant mouse strains while it was significantly abundant in the ileum microbiota of infected susceptible OcB/Dem strains. Those divergent results are because of the distinction in the diversity of faecal microbiota and the microbiota of the small intestine and the large intestine.

Parasites *Leishmania infantum* (Cavallone et al. 2022; de Lima et al. 2021; Passos et al. 2020) and *Leishmania* (*Viannia*) *braziliensis* (Santos et al. 2021) were detected in gastrointestinal tract of hamsters, whereas presence of *L. donovani* was observed in the intestinal tract of mice and hamsters (Lewis et al. 2020) and in the human duodenum (Chattopadhyay et al. 2020). There is no literary reports about the detection of *L. major* in the intestinal tract of mice, and it was out of the scope of our study to analyse the parasitic dissemination to the ileum or the colon. However, we cannot exclude that *L. major* parasites could be present in the ileum or colon and can influence the microbiome composition.

PICRUSt was used to obtain the functional composition and determine differences in the intestinal microbiota in infected resistant and infected susceptible mice (Supplemental Figs. S1 and S2, Supplemental Tables S6–S8). PICRUSt cluster analysis showed significant differences between the ileum and colon. In the ileum function, there were large distinctions between clusters of infected resistant and infected susceptible mice which lead to suggesting that the ileum microbiota is the most affected intestinal part by *Leishmania* infection. The functional prediction analysis revealed richness in the intestinal microbiota with many microbial functional genes related to “metabolism”, “environment information processing”, “genetic information processing”, “human diseases” and “cellular processing”.

It was inferred that the ileum microbiome of resistant OcB/Dem strains was significantly rich in genes related to xenobiotic biodegradation and metabolism (metabolism of xenobiotics by cytochrome P450 in KEGG level 3). Intestinal microbiota represents a site for xenobiotic metabolism (Björkholm et al. 2009; Toda et al. 2009; Li et al. 2017; Collins and Patterson 2020). The gut microbiome regulates the metabolic outcomes of xenobiotic and host gene expression of CYP450s (Collins and Patterson 2020). Experimental studies have investigated the impact of leishmaniasis on drug metabolism by decreasing cytochrome P450 (CYP) levels. An early study has suggested that infection with *L. donovani* affects the xenobiotic-metabolising enzymes of mice liver (Coombs et al. 1990). It was reported that the phenotypic activities of CYP3A4 and CYP2C19 were significantly reduced in Brazilian patients during the acute phase of visceral leishmaniasis (Lanchote et al. 2015). Decreased xenobiotic metabolism function leads to an alteration of drug clearance rates, which would have severe implications for individuals infected with *Leishmania*. Another study has revealed that nitric oxide (NO) mediates the impairment of cytochrome P450-dependent metabolism in the liver of hamsters infected with *L. donovani* (Samanta et al. 2003).

Genes related to amino acid metabolism function were upregulated in the ileum microbiome of infected resistant mice in all model strains. Some amino acids such as arginine, asparagine and tryptophan are considered mediators of metabolic cross-talk between the host and pathogen (Ren et al. 2018). The supplementation of glutamine to mice infected with *L*. *donovani* may act as a promising adjuvant during miltefosine treatment to improve anti-*Leishmania* immune response by considerably decreasing the parasite charge (Ferreira et al. 2020). The L-arginine metabolic pathway was realised to be required in the regulation of iNOS-mediated parasite killing and polyamine-mediated parasite growth. Also, amino acids are involved in the regulation of immune cells during leishmaniasis (Wanasen and Soong 2008).

The abundance of genes involved in “carbohydrate metabolism” and “glycan biosynthesis and metabolism” was significantly higher in the gut microbiome of the infected susceptible mice. It was determined that *L. major* promastigotes induce the macrophages to promote anaerobic glycolysis and cause the accumulation of cholesterol (Rabhi et al. 2012). The observations of Bodhale et al. (2018) suggest an association between cytokine secretion profile, *Leishmania* susceptibility and the expression of different enzymes of the glycolytic pathway in the spleen of *L. donovani*-infected mice. A recent study has inferred that diet-induced obesity reduces the resistance of C57BL/6 mice to *L*. *major* infection (Martins et al. 2020).

This study assessed differences in cutaneous leishmaniasis outcomes in the colon and ileum using different experimental mice models. Bacterial composition and diversity of intestinal microbiota were analysed in susceptible and resistant leishmaniasis mice models. *L. major* infection altered the ileum microbiome composition widely; however, no significant changes in the colon microbiome have been observed. Moreover, the results indicated that the host genetic make-up has an important impact on shaping and modulation of gut microbiome composition. In addition, a large dissimilarity in beta diversity of the colon microbiome was noticed between all mice strains. Furthermore, the present study revealed biomarkers in the gut microbiome that were linked to susceptibility or resistance to the infection with *L. major*. Rikenellaceae was the common biomarker correlated with susceptibility in the colon microbiota of all infected susceptible mice strains. Several biomarkers were determined in the ileum microbiota of infected resistant OcB/Dem mice, and Moraxellaceae was the dominant biomarker. Besides, potential gene functions in the gut microbiome of infected mice were highlighted. Xenobiotic biodegradation and metabolism and amino acid metabolism pathways were principally related to the ileum microbiome of infected resistant strains, whereas genes associated with carbohydrate metabolism and glycan biosynthesis and metabolism were significantly higher in the gut microbiome of the infected susceptible mice. Determination of biomarkers and prediction of possibly implicated functional pathways related to the susceptible or resistant models may provide useful insight into pre-emptive microbiome-based medicine and the development of novel anti-leishmanial drugs by identifying new targets.

## Supplementary Information

Below is the link to the electronic supplementary material.

Supplementary file1 (PDF 1642 KB)

Supplementary file2 (XLSX 695 KB)

## Data Availability

Sequences were deposited into the SRA database of NCBI under accession number: PRJNA973043.
