# Supplementary material for: Effects of Leishmania major infection on the gut microbiome of resistant and susceptible mice
Source: Appl Microbiol Biotechnol. 2024 Jan 19;108:145. doi: 10.1007/s00253-024-13002-y (PMC10799115; doi:10.1007/s00253-024-13002-y)
Supplement: Supplementary file 1 [file 253_2024_13002_MOESM1_ESM.pdf]

Applied Microbiology and Biotechnology: Supplemental Material

Effects of *Leishmania major* infection on the gut microbiome of resistant and susceptible mice

Jakub Mrázek [1], Lucie Mrázková [2,3], Chahrazed Mekadim [1*], Taťána Jarošíková [3], Imtissal Krayem [2], Yahya Sohrabi[2,5,6], Peter Demant [4], Marie Lipoldová [2,3,5]

[1] Laboratory of Anaerobic Microbiology, Institute of Animal Physiology and Genetics of the Czech Academy of Sciences, Videnska 1083, 142 20, Prague, Czech Republic.

[2] Laboratory of Molecular and Cellular Immunology, Institute of Molecular Genetics of the Czech Academy of Sciences, Videnska 1083, 142 20, Prague, Czech Republic.

[3] Faculty of Biomedical Engineering, Czech Technical University in Prague, namestí Sitna 3105, 272 01 Kladno, Czech Republic.

[4] Department of Molecular and Cellular Biology, Roswell Park Comprehensive Cancer Center, Buffalo, New York 14263, USA.

[5] Department of Medical Genetics, 3rd Faculty of Medicine, Charles University, Ruská 87, 100 00 Prague 10, Czech Republic

[6] Department of Cardiology I-Coronary and Peripheral Vascular Disease, Heart Failure, University Hospital Münster, Westfälische Wilhelms-Universität, Münster, Germany.

*** Correspondence:**
Chahrazed Mekadim
mekadim@iapg.cas.cz
Phone : +420 267 090 501
Fax: +420 267 090 500
Address: Laboratory of Anaerobic Microbiology, Institute of Animal Physiology and Genetics of the Czech Academy of Sciences, Videnska 1083, 142 20, Prague, Czech Republic

Supplementary figure and legends

Figure S1

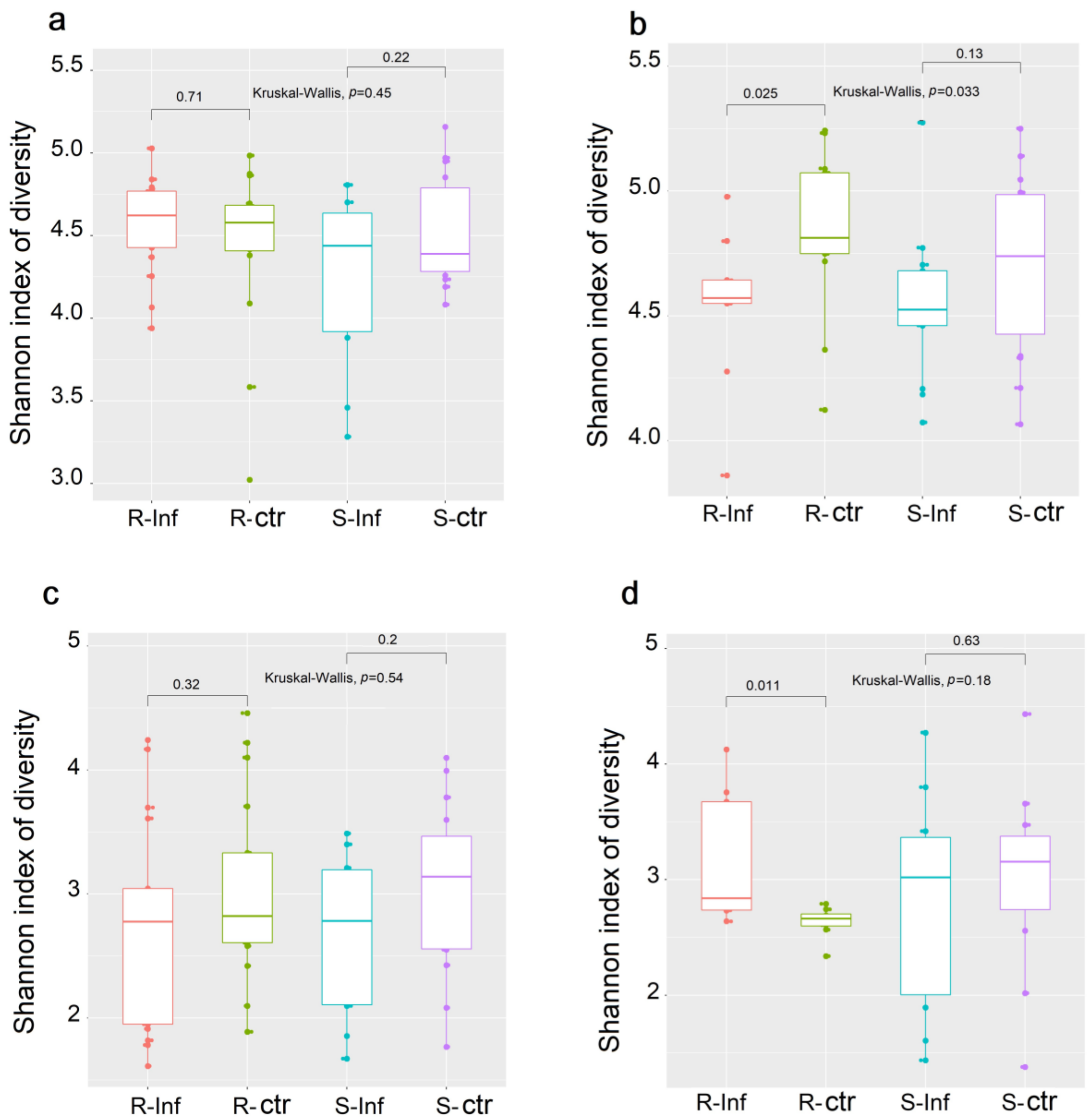

Fig. S1 Microbiota alpha diversity (shannon index) among different groups (susceptibles/ resistants) of infected or non-infected mice in: a) colon of CcS/Dem, b) colon of OcB/Dem, c) ileum of CcS/Dem and d) ileum of OcB/Dem. (p-value ≤ 0.05) was considered statistically significant. [R_Inf: infected resistant, R_ctr: resistant control (non-infected), S_Inf: infected susceptible, S_ctr: susceptible control (non-infected)].

Figure S2

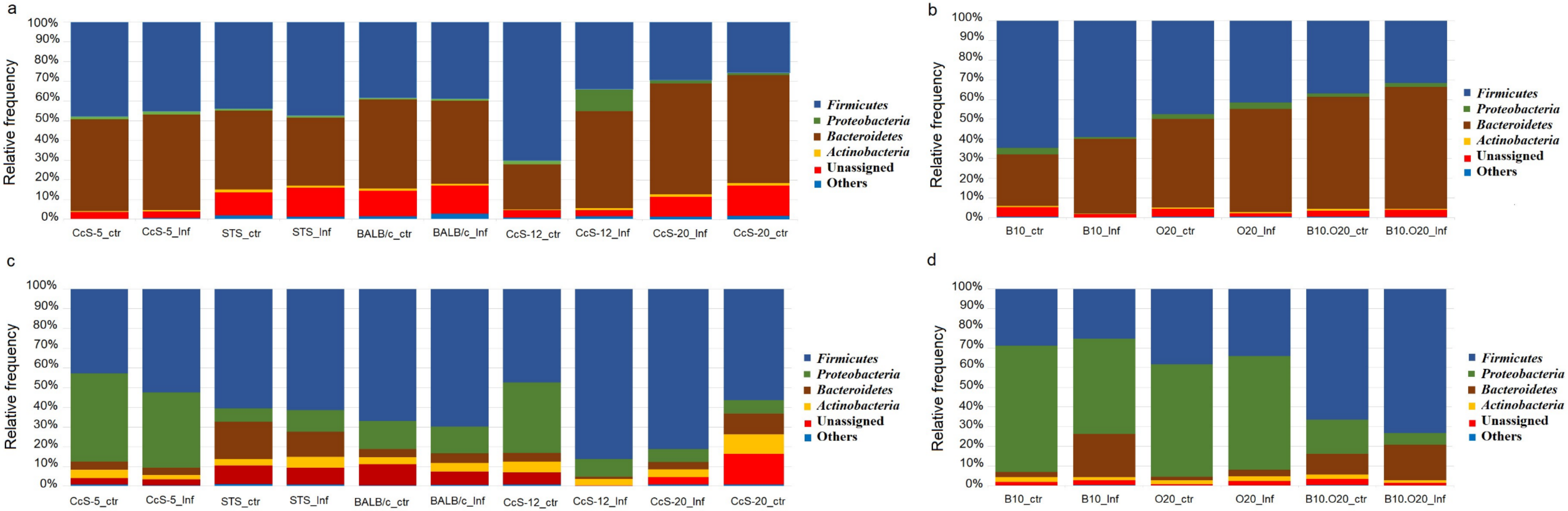

Fig. S2 Relative abundance of the microbial population at the phylum level among different groups (susceptibles/ resistants) of infected or non-infected mice in: a) colon of CcS/Dem, b) colon of OcB/Dem, c) ileum of CcS/Dem and d) ileum of OcB/Dem. [Inf: infected, ctr: control (non-infected)

Figure S3

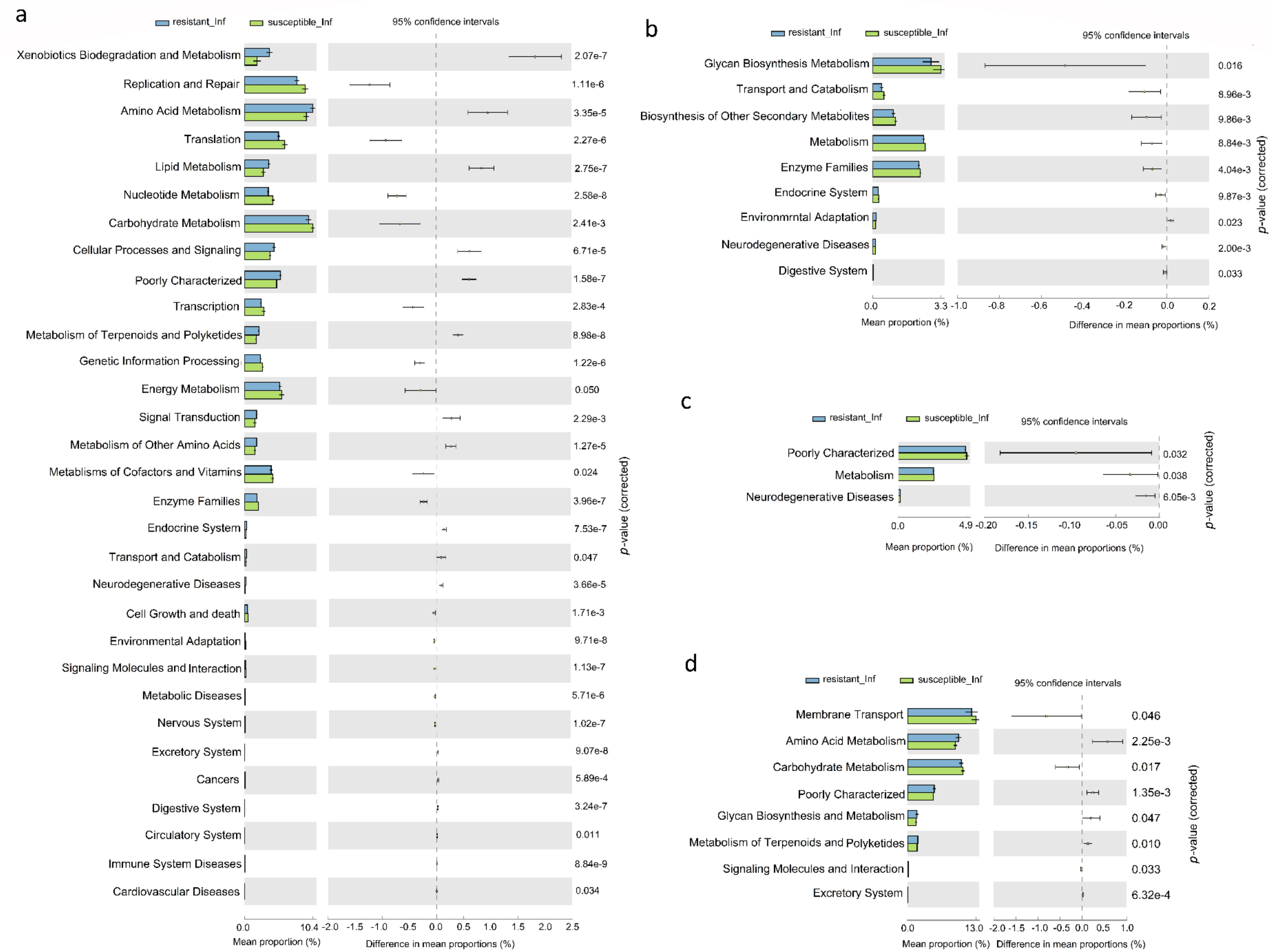

Fig. S3 Extended error bar plot showing the mean proportion (%) of significantly different predicted functional categories at second-level KEGG pathway between infected susceptible mice (in green) and infected resistant mice (in blue) in a) ileum of OcB/Dem, b) colon of OcB/Dem, c) ileum of CcS/Dem and d) colon of CcS/Dem. The values on the right show the corrected *p*-values that were derived from a Welch's *t*-test with the confidence interval method of Welch's inverted adjustment of 0.95 (*p*-value ≤ 0.05) was considered statistically significant. [Resistant_Inf: infected resistant, Susceptible_Inf: infected susceptible].

Figure S4

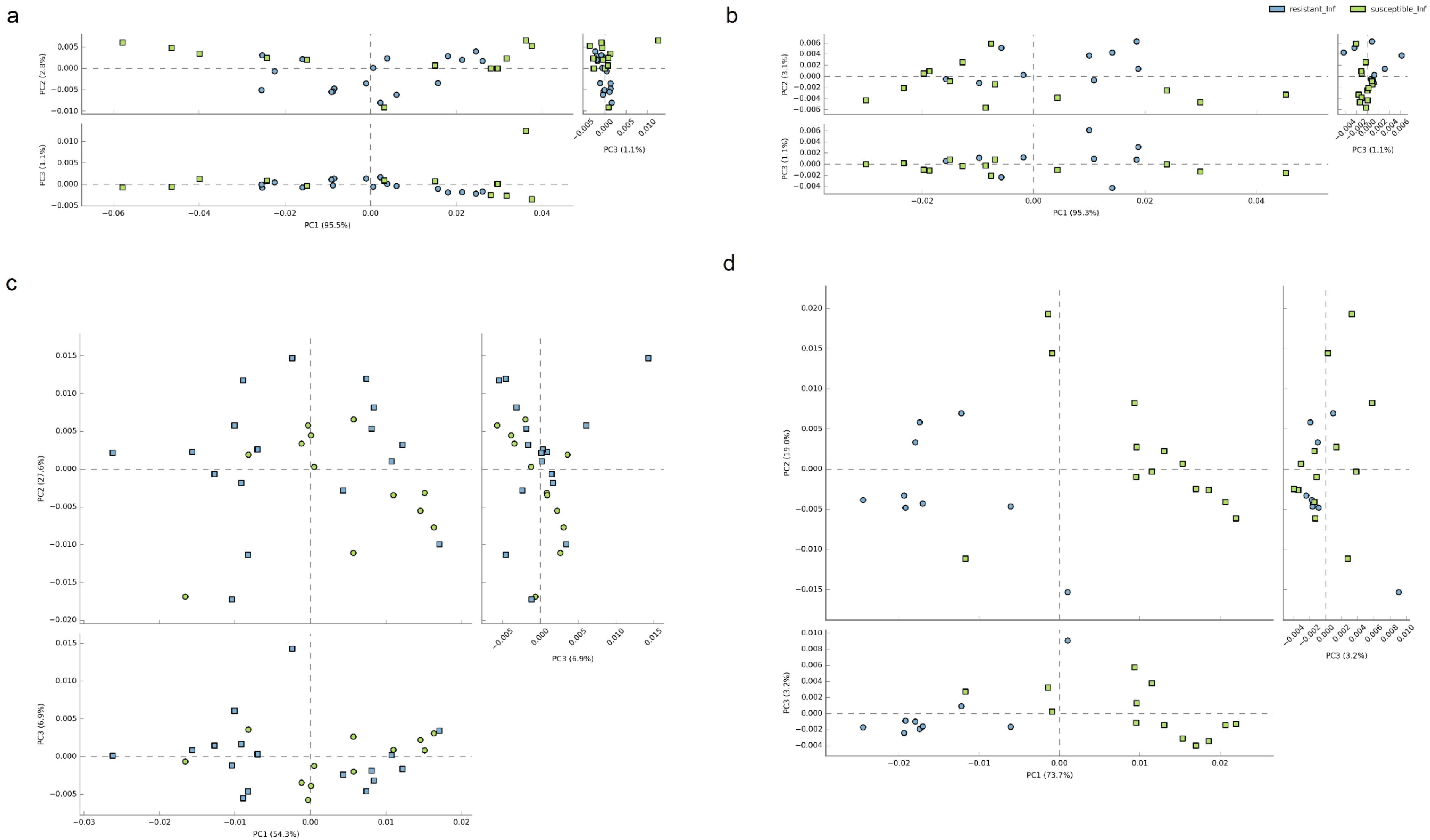

Fig. S4 Principal component analysis (PCA) plot of distances between second-level KEGG pathway profiles of infected susceptible mice (in green) and infected resistant (in blue) using the STAMP software based on Non-corrected Welch's *t*-test type two-sided, with the confidence interval (CI) method of Welch's inverted adjustment of 0.95 (p-value ≤ 0.05) was considered statistically significance. a) colon of CcS/Dem, b) colon of OcB/Dem, c) ileum of CcS/Dem and d) ileum of OcB/Dem. [Resistant_Inf: infected resistant, Susceptible_Inf: infected susceptible].
